# Association of family history of schizophrenia and clinical outcomes in individuals with eating disorders

**DOI:** 10.1017/S0033291721001574

**Published:** 2023-01

**Authors:** Ruyue Zhang, Ralf Kuja-Halkola, Andreas Birgegård, Henrik Larsson, Paul Lichtenstein, Cynthia M. Bulik, Sarah E. Bergen

**Affiliations:** 1 Department of Medical Epidemiology and Biostatistics, Karolinska Institutet, Stockholm, Sweden; 2 School of Medical Sciences, Örebro University, Örebro, Sweden; 3 Department of Psychiatry, University of North Carolina at Chapel Hill, Chapel Hill, USA; 4 Department of Nutrition, University of North Carolina at Chapel Hill, Chapel Hill, USA

**Keywords:** Clinical features, comorbidity, eating disorders, family history, schizophrenia

## Abstract

**Background:**

Familial co-aggregation studies of eating disorders (EDs) and schizophrenia reveal shared genetic and environment factors, yet their etiological and clinical relationship remains unclear. We evaluate the influence of schizophrenia family history on clinical outcomes of EDs.

**Methods:**

We conducted a cohort evaluation of the association between family history of schizophrenia and ED clinical features, psychiatric comorbidities, and somatic and mental health burden in individuals born in Sweden 1977–2003 with anorexia nervosa (AN) or other EDs (OED: bulimia nervosa, binge-eating disorder, and ED not otherwise specified).

**Results:**

Of 12 424 individuals with AN and 20 716 individuals with OED, 599 (4.8%) and 1118 (5.4%), respectively, had a family history of schizophrenia (in up to third-degree relatives). Among individuals with AN, schizophrenia in first-degree relatives was significantly associated with increased comorbid attention-deficit/hyperactivity disorder (ADHD) [HR(95% CI) 2.26 (1.27–3.99)], substance abuse disorder (SUD) [HR (95% CI) 1.93 (1.25–2.98)], and anxiety disorders [HR (95% CI) 1.47 (1.08–2.01)], but higher lowest illness-associated body mass index (BMI) [1.14 kg/m^2^, 95% CI (0.19–2.10)]. Schizophrenia in any relative (up to third-degree) in AN was significantly associated with higher somatic and mental health burden, but lower ED psychopathology scores [−0.29, 95% CI (−0.54 to −0.04)]. Schizophrenia in first-degree relatives in individuals with OED was significantly associated with increased comorbid ADHD, obsessive-compulsive disorder, SUD, anxiety disorders, somatic and mental health burden, and suicide attempts.

**Conclusions:**

We observed different patterns of ED-related outcomes, psychiatric comorbidity, and illness burden in individuals with EDs with and without family histories of schizophrenia and provide new insights into the diverse manifestations of EDs.

## Introduction

As the strongest single indicator of individual schizophrenia risk, family history of schizophrenia is also associated with higher treatment resistance (Kowalec et al., 2019; Mortensen et al., 1999), as well as greater risk for several psychiatric disorders (DeVylder & Lukens, 2013), including eating disorders (EDs). Recent large-scale studies revealed substantial familial co-aggregation of schizophrenia and EDs, indicating shared genetic and environmental factors between these conditions (Zhang et al., 2020). These observations are consistent with the role of familial factors for schizophrenia in the development of EDs. However, little is known about the influence of family history of schizophrenia on the clinical features of EDs.

A familial liability to schizophrenia could influence ED manifestations in a variety of ways. Objectively, low body mass index (BMI) is one of the diagnostic criteria for anorexia nervosa (AN) (American Psychiatric Association, 2000), and both AN and schizophrenia are negatively genetically correlated with BMI (Bulik-Sullivan et al., 2015; Duncan et al., 2017; Ikeda et al., 2018; Watson et al., 2019). However, individuals with schizophrenia commonly have obesity (Vancampfort et al., 2016), possibly due to side effects of antipsychotic medications (De Hert, Detraux, van Winkel, Yu, & Correll, 2011; Malan-Muller et al., 2016). It is, therefore, unclear whether a family history of schizophrenia might influence BMI in individuals with EDs.

Age at onset for EDs may also vary according to whether schizophrenia is present in family members. Increased exposure to genetic and environmental risk factors including family history of depression, inadequate parental control, etc., is associated with earlier ages of onset for bulimia nervosa (Schmidt, Hodes, & Treasure, 1992). In addition, having a family member with schizophrenia could elevate both genetic and environmental risk, potentially driving ages of onset earlier. Symptom patterns in EDs could also be influenced by a family history of schizophrenia. An assessment of disordered-eating behaviors in individuals with schizophrenia revealed high scores for restraint, uncontrolled, and emotional eating (Kouidrat et al., 2018). Individuals with EDs who have schizophrenia running in their family may show elevated scores in these domains compared to those with no family history of schizophrenia.

Psychiatric comorbidities are very common in EDs [e.g. major depressive disorder (MDD) 36–68%, anxiety disorders 37–56%, obsessive-compulsive disorder (OCD) 22–32%] (Blinder, Cumella, & Sanathara, 2006; O'Brien & Vincent, 2003). Although comorbid schizophrenia is less commonly observed clinically compared to other psychiatric comorbidities in EDs, it is in actuality 6–7× more likely in individuals with EDs than the general population (Zhang et al., 2020). Recent studies provide evidence of genetic overlap between schizophrenia and AN (genetic correlation, *r*_g_ = 0.19–0.29) (Bulik-Sullivan et al., 2015; Duncan et al., 2017; Watson et al., 2019), which has been further corroborated by large-scale studies demonstrating familial liability between schizophrenia and AN as well as other EDs (OED) (Zhang et al., 2020). Comorbid psychiatric diagnoses often complicate case conceptualization and treatment planning in clinical work for EDs. For example, earlier research reported cases with ‘multi-impulsive bulimia’ exhibiting more general psychopathology including anxiety, depression, psychoticism, etc., and higher comorbidity burden. However, the impact of family history of schizophrenia on the clinical presentation of EDs has not been investigated in previous studies (Fichter, Quadflieg, & Rief, 1994).

In addition to psychiatric comorbidity, EDs and schizophrenia are both phenotypically and genetically associated with multiple somatic complications and illnesses (Bulik-Sullivan et al., 2015; Herpertz-Dahlmann, 2015; Olguin et al., 2017; Watson et al., 2019; Westmoreland, Krantz, & Mehler, 2016). These illnesses contribute substantially to the overall morbidity and early mortality for EDs and schizophrenia (Crump, Winkleby, Sundquist, & Sundquist, 2013; Himmerich et al., 2019; Schoepf, Uppal, Potluri, & Heun, 2014). Again, the impact of genetic liability for schizophrenia on cumulative somatic and mental health burden in EDs has not previously been investigated.

The aim of this study was to explore the influence of family history of schizophrenia (as an indicator of genetic liability for schizophrenia) on clinical features and comorbidities in individuals with EDs. Specifically, we investigated the associations between schizophrenia family history and age of EDs onset, ED symptoms, and other clinical characteristics in addition to estimating the risk for psychiatric comorbidities, cumulative somatic and mental health burden, and suicide attempts in individuals with EDs with and without a family history of schizophrenia.

## Methods

### Data source

The data were derived from Swedish national registers linked by unique personal identification numbers (Ludvigsson et al., 2016). We obtained sex and birth year information from the Total Population Register (TPR), information regarding stillbirths and congenital malformations from the Medical Birth Register, migration data via the Migration Register, and dates and causes of death from the Cause of Death Register (CDR) (Axelsson, 2003; Ludvigsson et al., 2016). Both inpatient and specialist outpatient care contacts from across Sweden were obtained from the National Patient Register [NPR, based on the International Classification of Diseases (ICD)] (Ludvigsson et al., 2011). We also derived ED diagnoses and ED-related clinical features [e.g. BMI, Global Assessment of Functioning (GAF) score, EDE-Q subscale and global scores] from the National Eating Disorder Quality Registers (Riksät and Stepwise) (Birgegard, Bjorck, & Clinton, 2010).

The use of these data has been approved by the regional ethical review board in Stockholm, Sweden (Dnr 2013/862-31/5). No individuals in the study population were identifiable at any time.

### Study population and study design

The study population included all individuals born between 1 January 1977 and 31 December 2003 registered in the Swedish TPR, who were alive and residing in Sweden on their sixth birthday and had ever received diagnoses of AN and/or OED within the follow-up period. Individuals were followed from their sixth birthday until death, emigration from Sweden, or 31 December 2013, whichever came first. For the specific diagnostic codes used, see online Supplementary Table S1. After exclusions for stillbirths, congenital malformations, immigration, death or emigration before their sixth birthday, and incomplete biological parental information, 12 424 individuals with AN and 20 716 individuals with OED remained. Individuals could be included in both AN and OED categories if they received both diagnoses.

### Assessment of family history of schizophrenia

Family history of schizophrenia was identified via linkage with the Swedish Multi-Generation Register and categorized into two levels: (1) first-degree relatives with schizophrenia, including any parent or full-sibling; (2) any relative with schizophrenia, including any parent, full-sibling, half-sibling, grandparent, uncle, aunt, or cousin. Individuals with schizophrenia were defined as having received at least two inpatient or outpatient treatment contacts for schizophrenia or schizoaffective disorders from the Swedish NPR and the CDR. The ICD diagnostic codes for schizophrenia are outlined in online Supplementary Table S1.

### Assessment of outcomes

Outcomes of interest included clinical features of EDs, psychiatric comorbidities, and cumulative somatic and mental health burden. The clinical features of EDs were: age at first ED diagnosis, the lowest illness-associated BMI, the lowest recorded GAF score, and the highest recorded EDE-Q scores. Psychiatric comorbidities included disorders demonstrating prior shared genetic risk with AN and common psychiatric comorbidities in EDs: attention-deficit/hyperactivity disorder (ADHD), substance abuse disorder (SUD), autism spectrum disorder (ASD), MDD, OCD, and anxiety disorders (Kaye, Bulik, Thornton, Barbarich, & Masters, 2004; Spindler & Milos, 2007; Swinbourne & Touyz, 2007; Wade, Bulik, Neale, & Kendler, 2000; Watson et al., 2019). Cumulative somatic and mental health burden included the total number of all available somatic and psychiatric diagnoses received, the total number of all available unique diagnoses received, and the total number of suicide attempts recorded.

Age at first AN and/or OED diagnosis was calculated based on the date of the first treatment contact for AN and/or OED from the NPR and ED quality registers. The lowest BMI and GAF score, and the highest EDE-Q scores (restraint, eating concern, weight concern, shape concern, and global score) in records for each individual were obtained from ED quality registers (Riksät and Stepwise) (Birgegard et al., 2010). Psychiatric comorbidities and the date of initial diagnosis for each disorder were identified from the NPR and CDR using the ICD diagnostic codes presented in online Supplementary Table S1. The total number of somatic and psychiatric diagnoses received included all in- and outpatient ICD records with both primary and secondary diagnoses available in the dataset. Total unique diagnoses were counted from total diagnoses after removing duplicated records. Total suicide attempts were identified from the NPR and the CDR using the ICD diagnostic codes presented in online Supplementary Table S1. All cumulative outcomes including total diagnoses, unique diagnoses, and suicide attempts were counted within each time period (before age 12, age 12 up to age 19, age 19 up to age 26, age 26 and up) for each individual, respectively.

### Statistical analyses

For all analyses, data management was performed using SAS, version 9.4 (SAS Institute, Inc.), and all analyses were performed using R, version 3.4.3.

Linear regressions were used for age at first diagnosis, BMI, GAF score, and EDE-Q scores including restraint, eating concern, shape concern, weight concern, and global scores adjusting for birth year and sex. Regression coefficients with 95% confidence intervals (CIs) and corresponding *p* values were reported. Cluster-robust (sandwich) estimators, clustered on family identification numbers, were used to control for dependencies between individuals in the same family.

Cox regressions with age as the underlying time-scale were used for psychiatric comorbidities including ADHD, ASD, OCD, MDD, SUD, and anxiety disorders while adjusting for sex and birth year. Individuals were followed from their sixth birthday until death, emigration from Sweden, or 31 December 2013, whichever came first. Hazard ratios (HRs) with 95% CIs were reported as risk estimates for psychiatric comorbidities comparing individuals with EDs with and without a family history of schizophrenia. Cluster-robust (sandwich) estimators using family identification numbers were calculated to control for non-independencies between individuals in the same family.

Time-split Poisson regressions were used for cumulative somatic and mental health burden including total diagnoses, total unique diagnoses, and total suicide attempts with adjustments for birth year, sex, and different time periods (before age 12, age 12 up to age 19, age 19 up to age 26, age 26 and up). Incidence rate ratios (IRRs) with 95% CIs were calculated. We used a cluster-robust (sandwich) estimator for standard error calculation, where clusters were identified via individual identification numbers to correct for non-independence.

In order to investigate whether following individuals for the outcome from age 6 regardless of when they received the ED diagnosis would impact the results, we performed further sensitivity analyses in which the outcomes, including psychiatric comorbidities and cumulative somatic and mental health burden, were measured after the first ED diagnosis using the same regression models as above. In addition, ED onset precedes the date of a first ED diagnosis by an unknown duration. We performed a sensitivity analysis examining the period preceding the ED diagnosis, to assess whether family history of schizophrenia exerts any influence prior to the date of ED diagnosis.

Furthermore, the recorded health care occasions only included a subset of all occasions, namely those with a non-random subset of ICD codes occurring as a primary or secondary diagnosis, including all occasions with an ED code. For these occasions, all ICD-coded diagnoses were recorded in the dataset. Therefore, in addition to counting cumulative lifetime diagnoses, we also performed a sensitivity analysis only counting those diagnoses observed at the same date together with each ED diagnosis.

## Results

Descriptive characteristics and outcomes of interest are provided in Table 1. The study population comprised 12 424 individuals with AN and 20 716 individuals with OED identified from the Swedish national registers with a total follow-up of 230 323 person-years for AN and 395 585 person-years for OED. Of the identified individuals with AN, 94.2% were female and 93.7% of those with OED were female. A total of 70 (0.56%) individuals with AN and 133 (0.64%) individuals with OED also had received diagnoses of schizophrenia during the full follow-up period. The descriptive statistics for individuals with EDs and schizophrenia are shown in online Supplementary Table S2.

**Table 1.** Descriptive characteristics of the study population

	AN (*N* = 12 424)	OED (*N* = 20 716)
Sex		
Female (%)	11 704 (94.2)	19 420 (93.7)
Male (%)	720 (5.8)	1296 (6.3)
Birth year (mean ± s.d.)	1988.9 ± 5.7	1988.4 ± 5.8
Family history of schizophrenia (%)		
1st degree relatives	94 (0.76)	199 (0.96)
Any relative	599 (4.82)	1118 (5.40)
Age at first AN/OED diagnosis (mean ± s.d.)	17.97 ± 4.22	19.75 ± 4.86
BMI (mean ± s.d.)^a^	17.41 ± 2.71	20.33 ± 4.67
GAF score (mean ± s.d.)^b^	47.60 ± 13.45	49.17 ± 12.15
EDE-Q scores (mean ± s.d.)^c^		
Restraint	3.38 ± 1.85	3.42 ± 1.72
Eating concern	2.78 ± 1.50	3.04 ± 1.45
Weight concern	3.47 ± 1.74	3.84 ± 1.59
Shape concern	4.14 ± 1.74	4.49 ± 1.58
Global score	3.42 ± 1.56	3.68 ± 1.43
Schizophrenia	70 (0.56)	133 (0.64)
Psychiatric comorbidities (%)		
Anxiety disorders	3445 (27.7)	7515 (36.3)
OCD	923 (7.4)	1541 (7.4)
MDD	4426 (35.6)	8 895 (42.9)
ASD	430 (3.5)	838 (4.1)
ADHD	685 (5.5)	1826 (8.8)
SUD	1435 (11.6)	3144 (15.2)
Total follow-up years	230 322.6	395 585.1
Total diagnoses	472 181	842 343
Total unique diagnoses	201 317	378 949
Total suicide attempts	7580	14 777

AN, anorexia nervosa; OED, other eating disorders; s.d., standard deviation; BMI, body mass index; GAF scores, Global Assessment of Functioning scores; EDE-Q scores, Eating Disorders Examination Questionnaire scores; OCD, obsessive-compulsive disorder; MDD, major depressive disorder; ASD, autism spectrum disorder; ADHD, attention-deficit/hyperactivity disorder; SUD, substance abuse disorders.

a There were 7112 individuals with AN with BMI information, and 9667 individuals with OED with BMI information.

b There were 6656 individuals with AN with GAF score information, and 8905 individuals with OED with GAF score information.

c There were 3556 individuals with AN with EDEQ information, and 5515 individuals with OED with EDE-Q information.

Among all individuals with AN, 0.76% had a first-degree family history of schizophrenia and 4.82% had at least one relative (including up to third-degree relatives) with schizophrenia. Among those with OED, 0.96% had a first-degree family history of schizophrenia and 5.40% individuals had a family history of schizophrenia including up to third-degree relatives.

The average age at first ED diagnosis was 17.9 years for AN and 19.8 years for OED. The most prevalent psychiatric comorbidities during the full follow-up period were MDD and anxiety disorders in both AN and OED. Among identified individuals with available information, the mean highest EDE-Q global score was 3.42 in AN and 3.68 in OED. In individuals with AN, the average lowest illness-related BMI was 17.41 kg/m^2^. Other characteristics and outcomes including birth year, GAF score, total diagnoses, total unique diagnoses, and total suicide attempts are described in detail in Table 1.

The estimated differences with 95% CIs and corresponding *p* values for age at first ED diagnosis, BMI, GAF score, and EDE-Q scores among individuals with and without a family history of schizophrenia are presented in Table 2. The age at first diagnosis for both AN and OED did not differ in relation to family history of schizophrenia. Among individuals with AN, family history of schizophrenia in first-degree relatives was associated with higher lowest illness-associated BMI of 1.14 kg/m^2^ (*p* = 0.018). No statistically significant association was observed for BMI and family history of schizophrenia in OED. Notably, among individuals with AN, those with at least one relative (including up to third-degree relatives) with schizophrenia had significantly lower EDE-Q scores including the global score and all subscale scores compared to those without any family history of schizophrenia. Similar trends were also detected for lower EDE-Q scores in individuals with OED who had a family history of schizophrenia. However, there was no statistically significant evidence for these differences in individuals with OED. No significant association was observed between GAF scores and family history of schizophrenia in both AN and OED.

**Table 2.** Linear regression of ED clinical features and family history of schizophrenia

		1st-degree relatives with schizophrenia		Any relative with schizophrenia	
ED types		Regression coefficient (95% CI)	*p* value	Regression coefficient (95% CI)	*p* value
AN	Age at first diagnosis	0.21 (−0.60 to 1.02)	0.62	−0.14 (−0.44 to 0.17)	0.37
	BMI^a^	**1.14 (0.19 to 2.10)**	**0.018**	−0.26 (−0.57 to 0.04)	0.094
	EDE-Q scores^b^				
	Restraint	0.02 (−0.64 to 0.68)	0.954	**−0.32 (−0.61** to **−0.03)**	**0.031**
	Eating concern	−0.29 (−0.80 to 0.21)	0.25	**−0.23 (−0.46** to **−0.0004)**	**0.049**
	Weight concern	−0.24 (−0.87 to 0.39)	0.448	**−0.29 (−0.56** to **−0.01)**	**0.039**
	Shape concern	−0.26 (−0.85 to 0.32)	0.378	**−0.37 (−0.65** to **−0.10)**	**0.008**
	Global score	−0.21 (−0.75 to 0.33)	0.448	**−0.29 (−0.54** to **−0.04)**	**0.021**
	GAF^c^	−1.25 (−4.90 to 2.41)	0.503	−1.39 (−2.89 to 0.11)	0.070
OED	Age at first diagnosis	−0.20 (−0.76 to 0.36)	0.485	−0.04 (−0.26 to 0.18)	0.707
	BMI^a^	−0.15 (−0.96 to 0.67)	0.720	−0.27 (−0.65 to 0.11)	0.168
	EDE-Q scores^b^				
	Restraint	0.09 (−0.39 to 0.56)	0.720	−0.14 (−0.34 to 0.06)	0.163
	Eating concern	−0.15 (−0.54 to 0.25)	0.461	−0.08 (−0.24 to 0.09)	0.350
	Weight concern	−0.09 (−0.60 to 0.42)	0.721	−0.07 (−0.26 to 0.11)	0.442
	Shape concern	−0.20 (−0.67 to 0.26)	0.392	−0.18 (−0.37 to 0.005)	0.056
	Global score	−0.10 (−0.51 to 0.31)	0.632	−0.11 (−0.28 to 0.06)	0.198
	GAF^c^	−0.39 (−3.17 to 2.40)	0.786	−1.00 (−2.17 to 0.17)	0.094

ED, eating disorder; CI, confidence interval; AN, anorexia nervosa; OED, other eating disorders; BMI, body mass index; GAF scores, Global Assessment of Functioning scores; EDE-Q scores, Eating Disorders Examination Questionnaire scores.

**Bold font** indicates statistical significance, p<0.05.

a There were 7112 individuals with AN with BMI information, and 9667 individuals with OED with BMI information.

b There were 3556 individuals with AN with EDEQ information, and 5515 individuals with OED with EDE-Q information.

c There were 6656 individuals with AN with GAF score information, and 8905 individuals with OED with GAF score information.

The prevalences of psychiatric comorbidities during the full follow-up period in individuals with and without a family history of schizophrenia are shown in online Supplementary Table S3. The number and proportion of individuals who received diagnoses for comorbid psychiatric disorders before/after ED diagnoses are shown in online Supplementary Table S4. The majority of the individuals with comorbid psychiatric diagnoses received them after AN (60–70%) or OED diagnoses (50–60%).

Results from the Cox models examining the risks for psychiatric comorbidities associated with family history of schizophrenia are presented in Table 3. A family history of schizophrenia in first-degree relatives was associated with an increased risk for comorbid ADHD, SUD, and anxiety disorders in both AN [HR (95% CI) ADHD = 2.25 (1.27–3.98); SUD = 1.93 (1.26–2.98); anxiety disorders = 1.47 (1.08–2.01)] and OED [ADHD = 1.59 (1.09–2.31); SUD = 1.45 (1.08–1.96); anxiety disorders = 1.36 (1.11–1.67)]. Elevated comorbid OCD was found to be associated with a first-degree family history of schizophrenia in OED [1.59 (1.08–2.36)], but not AN [1.01 (0.51–2.01)]. No significant increased risk was observed for MDD or ASD in either AN or OED with a first-degree family history of schizophrenia. The HRs for psychiatric comorbidities were observed to be associated with the degree of relatedness divided into first-, second-, and third-degree relatives (online Supplementary Table S5). There was a tendency for HRs for all psychiatric comorbidities associated with family history of schizophrenia to be attenuated with decreasing genetic relatedness in individuals with OED.

**Table 3.** The hazard ratios (HRs) of psychiatric comorbidities among individuals with EDs with schizophrenia family history

		1st-degree relatives with schizophrenia		Any relative with schizophrenia	
ED types		HR	95% CI	HR	95% CI
AN	Anxiety disorders	**1.47**	**(1.08–2.01)**	1.12	(0.96**–**1.30)
	MDD	1.29	(0.95**–**1.75)	1.11	(0.97**–**1.26)
	OCD	1.01	(0.51**–**2.01)	0.91	(0.67**–**1.24)
	ASD	1.67	(0.75**–**3.71)	1.12	(0.75**–**1.69)
	ADHD	**2.26**	(**1.27–3.99**)	1.06	(0.76**–**1.48)
	SUD	**1.93**	(**1.25–2.98**)	**1.30**	(**1.05–1.60**)
OED	Anxiety disorders	**1.36**	(**1.11–1.67**)	**1.13**	(**1.03–1.24**)
	MDD	1.18	(0.96**–**1.44)	**1.10**	(**1.01–1.21**)
	OCD	**1.59**	(**1.08–2.36**)	0.98	(0.78**–**1.22)
	ASD	1.49	(0.84**–**2.65)	1.26	(0.95**–**1.65)
	ADHD	**1.60**	(**1.10–2.33**)	1.19	(0.99**–**1.44)
	SUD	**1.47**	(**1.09–1.98**)	1.09	(0.94**–**1.26)

ED, eating disorder; CI, confidence interval; AN, anorexia nervosa; OED, other eating disorders; OCD, obsessive-compulsive disorder; MDD, major depressive disorder; ASD, autism spectrum disorder; ADHD, attention-deficit/hyperactivity disorder; SUD, substance abuse disorders.

**Bold font** indicates statistical significance, p<0.05.

After splitting the follow-up period into time before (online Supplementary Table S7) and after ED diagnoses (online Supplementary Table S8), SUD remained significantly associated with first-degree family history of schizophrenia in AN both before and after AN diagnoses. ADHD and anxiety in AN, and OCD in OED, were only statistically significant after ED diagnosis, whereas ADHD, anxiety, and SUD in OED were only statistically significant before OED diagnoses. MDD was only statistically significant with first-degree family history of schizophrenia in AN and OED in the period before ED diagnoses.

The IRRs for cumulative measurements including total diagnoses, total unique diagnoses, and suicide attempts among individuals with a family history of schizophrenia are presented in Table 4. Among individuals with OED, those with a first-degree family history of schizophrenia received 26% more diagnoses and 24% more unique diagnoses on average compared to those without a first-degree family history of schizophrenia. Any family history of schizophrenia was also significantly associated with more diagnoses/unique diagnoses in both AN and OED. Notably, among individuals with OED, those with a first-degree family history of schizophrenia had significantly more suicide attempts (IRR = 1.70, 95% CI 1.05–2.76). The association between a first-degree family history of schizophrenia in AN and suicide attempts was almost as high (IRR = 1.61, 95% CI 0.83–3.14), albeit not statistically significant.

**Table 4.** The incidence rate ratios (IRRs) of cumulative somatic and mental health burden among individuals with EDs with schizophrenia family history

		1st-degree relatives with schizophrenia		Any relative with schizophrenia	
ED types		IRR	95% CI	IRR	95% CI
AN	Total diagnoses	1.11	(0.88–1.42)	**1.14**	(**1.00–1.29**)
	Total unique diagnoses	1.18	(0.96–1.46)	**1.11**	(**1.01–1.21**)
	Total suicide attempts	1.61	(0.83–3.14)	1.35	(0.89–2.05)
OED	Total diagnoses	**1.26**	(**1.07**–**1.48**)	**1.09**	**(1.00–1.19**)
	Total unique diagnoses	**1.24**	**(1.08**–**1.41**)	**1.07**	**(1.01–1.14**)
	Total suicide attempts	**1.70**	**(1.05**–**2.76**)	1.12	**(**0.81–1.53)

ED, eating disorder; CI, confidence interval; AN, anorexia nervosa; OED, other eating disorders.

**Bold font** indicates statistical significance, p<0.05.

After splitting the follow-up period into time before (online Supplementary Table S9) and after (online Supplementary Table S10) ED diagnoses, the point estimates for cumulative outcomes remained stable, whereas the CIs widened. This led to non-statistical-significance for the majority of tests, which was likely due to insufficient power. When only analyzing the comorbid diagnoses recorded on the same date as ED diagnoses (online Supplementary Table S11), individuals who have any relative with schizophrenia had more unique diagnoses received together with AN.

## Discussion

Using a large population-based cohort of Swedish adolescents and adults, we assessed family history of schizophrenia in individuals with EDs in relation to health outcomes and comorbidities. Among 12 424 individuals with AN, and 20 716 individuals with OED, a small but clinically meaningful subset of individuals have co-occurring schizophrenia, and approximately 5% of individuals had family histories of schizophrenia (up to third-degree relatives). Clinical features of AN differed in those with family histories of schizophrenia, demonstrating greater psychiatric comorbidity burden, greater cumulative somatic and mental health burden, and increased suicide risk, but the severity of their EDs was somewhat less (e.g. lower EDE-Q scores and higher lowest illness-related BMI) than those without family history of schizophrenia. Interestingly, similar patterns were also detected in individuals with AN or OED who themselves had comorbid schizophrenia (online Supplementary Table S2).

Psychiatric comorbidities in individuals with EDs are common. The majority (55–97%) of individuals diagnosed with an ED also receive a diagnosis of at least one other psychiatric disorder, most commonly MDD, anxiety disorders, OCD, and SUD (Kaye et al., 2004; Spindler & Milos, 2007; Swinbourne & Touyz, 2007; Wade et al., 2000). Our findings suggest that individuals with AN or OED with first-degree family history of schizophrenia are more likely to have comorbid anxiety disorders, ADHD, and SUD. Family history of schizophrenia also has a positive association with OCD in individuals with OED, but not AN.

Furthermore, it is well known that EDs are associated with multiple somatic complications and illnesses (Herpertz-Dahlmann, 2015; Olguin et al., 2017; Westmoreland et al., 2016). Our observations suggest a more multi-morbid clinical picture in those individuals with AN or OED who have family histories of schizophrenia. That is, they have an array of psychiatric and somatic symptoms that cut across diagnostic categories, rather than presenting with more circumscribed and clearly defined EDs as seen in those without family histories of schizophrenia. These individuals may be particularly high on a general psychopathology factor, namely, a genetically driven propensity to develop multiple common psychopathologies (Caspi et al., 2014). Although lower EDE-Q scores indicate less ED pathology in people with a positive schizophrenia family history, they do not reflect the severity of the cumulative psychiatric and somatic illness burden.

Earlier research in EDs did point toward a multi-morbid presentation, initially termed ‘multi-impulsive bulimia’; however, this presentation tended to be severe and coupled with substance use disorders, self-harm, and borderline personality disorder. These individuals also exhibited more general psychopathology including anxiety, depression, psychoticism, etc., and higher comorbidity burden, although family history of schizophrenia was not investigated in those studies (Fichter et al., 1994).

A compatible etiological hypothesis is that varying genetic liability contributes to the differing clinical presentations in AN and OED with co-occurring schizophrenia or family history of schizophrenia. Recent genome-wide association studies indicate a high genetic correlation between schizophrenia and AN (Duncan et al., 2017; Watson et al., 2019). It is unknown whether part of this correlation is attributable to individuals in the studies who have family histories of the other disorder. Furthermore, it is conceivable (and eventually testable) that the extent of genetic loading for schizophrenia could manifest as a gradient in clinical presentations in AN or OED. This is implied by our findings in which the lowest EDE-Q scores, and the highest illness-associated BMI and psychiatric comorbidity prevalences are seen in individuals with co-occurring schizophrenia (online Supplementary Table S2) who presumably had the highest genetic loading for schizophrenia. Moreover, the differences in those with family histories of schizophrenia showed a decreasing gradient as the degree of relatedness of the family members with schizophrenia become more distant (online Supplementary Tables S5 and S6). Further studies on the impact of molecular genetic loading for schizophrenia on the course and outcome of EDs are needed to test the hypothesis.

The main strength of our investigation is the large-scale population-based cohort, which ensures greater statistical power and strengthens the reliability of the findings. The national coverage of all available psychiatric and somatic healthcare contacts, as well as detailed ED-related traits from national quality registers, provides a unique opportunity to deeply investigate the clinical manifestations of individuals with AN and OED. Moreover, family history information was not only limited to first-degree relatives, but also available up to the third-degree of relatedness, and this was derived from documented diagnoses and relationships rather than self-report.

Limitations to the study should be considered. Firstly, only a subset of the study population (27–57%) had available BMI, GAF score, or EDE-Q score information. These variables were recorded in the ED quality registers, and not all individuals identified from the Swedish NPR with EDs were included in the quality registers. However, the sample with available information is still one of the largest of its kind. Secondly, the diagnoses available in the dataset (472 181 for individuals with AN, 842 343 for individuals with OED) did not include all possible ICD diagnoses, which might lead to an underestimation of the cumulative somatic and mental health burden. Moreover, the available recorded health care occasions only included a non-random subset of ICD codes occurring as a primary or secondary diagnosis on all occasions. When counting the cumulative somatic and mental illness burden, there is a possibility that the diagnoses only co-occurring with the diagnoses explicitly included in our data might bias the estimates (i.e. cancer diagnoses are not generally included, but a hospitalization for cancer which also noted a concurrent ED would be captured in our data). The sensitivity analysis incorporating only the comorbid diagnoses recorded on the same date as an ED diagnosis weakened most associations, except for AN with any family history of schizophrenia. Thus, these results need to be viewed with caution (online Supplementary Table S11). Thirdly, since the oldest individuals in our sample were 36 at the end of follow-up, late-onset ED cases (and somatic and psychiatric comorbidities) might be underrepresented in our study population. Another limitation is the potential under-detection of diagnoses, since outpatient diagnosis records were only available after 2001. Furthermore, it is also impossible to capture recent diagnoses or clinical presentations later in life, beyond the follow-up period as some individuals may still develop EDs or schizophrenia later in life. However, this issue was partially addressed by the statistical models used.

Family history of schizophrenia is associated with notable differences in the clinical presentation of EDs. Significantly elevated risk for psychiatric comorbidities as well as cumulative somatic and mental health burden along with decreased severity of ED-related symptoms were observed in individuals with family history of schizophrenia. Whether a family history of schizophrenia will be a meaningful predictor of clinical response is a question worthy of consideration. For example, although olanzapine is known to be effective in the treatment of schizophrenia (Citrome, McEvoy, Todtenkopf, McDonnell, & Weiden, 2019), the latest multicenter trial reported a modest therapeutic effect of olanzapine compared with placebo on weight in adult outpatients with AN, but no significant benefit for psychological symptoms (Attia et al., 2019). A close examination of the outcome data reveals variable response across patients. Whether family history of schizophrenia could predict response to medications such as olanzapine that are known to be effective in the treatment of schizophrenia is unknown, but merits future study. Accordingly, although always advised, clinicians should take a detailed family history of psychiatric disorders including schizophrenia when assessing EDs as it may influence clinical presentations in EDs and potentially inform treatment decisions. Future studies will examine the impact of molecular genetic risk for schizophrenia on the course and outcome of EDs.

## Supplementary material

For supplementary material accompanying this paper visit https://doi.org/10.1017/S0033291721001574. click here to view supplementary material
