# Supplementary material for: Association of family history of schizophrenia and clinical outcomes in individuals with eating disorders
Source: Psychol Med. 2021 Apr 30;53(2):371–8. doi: 10.1017/S0033291721001574 (PMC9899560; doi:10.1017/S0033291721001574)
Supplement: Supplementary file 1 [file S0033291721001574sup.zip › S0033291721001574sup001.docx]

**Supplemental Material for**

Association of Family History of Schizophrenia and Clinical Outcomes in Individuals with Eating Disorders

Ruyue Zhang^a^, MSc, Ralf Kuja-Halkola^a^, PhD, Andreas Birgegård^a^, Henrik Larsson^a,b^, PhD, Paul Lichtenstein^a^, PhD, Cynthia M. Bulik^a,c,d^, PhD, Sarah E. Bergen^a^, PhD

^a^Department of Medical Epidemiology and Biostatistics, Karolinska Institutet, Stockholm, Sweden

^b^School of Medical Sciences, Örebro University, Örebro, Sweden

^c^Department of Psychiatry, University of North Carolina at Chapel Hill, Chapel Hill, USA

^d^Department of Nutrition, University of North Carolina at Chapel Hill, Chapel Hill, USA

| Table S1. Diagnostic codes used to define psychiatric conditions in the national patient register, the cause of death register, and the national eating disorder quality register | | | | |
| --- | --- | --- | --- | --- |
| Diagnoses | **ICD-8 (1973-1986)** | **ICD-9 (1987-1996)** | **ICD-10 (1997 to present)** | **ED quality registers^a^ (Riksät since 1999, Stepwise since 2005)** |
| AN | - | 307B | F50.0, F50.1 | DSM-IV-TR criteria 307.1; 307.50, criteria 1 and 2 |
| OED | - | 307F | F50.2, F50.3, F50.9 | DSM-IV-TR criteria 307.51; 307.50, criteria 3; or eating disorders not otherwise specified |
| Schizophrenia | 295.0-295.9 (except 295.5) | 295A-295H (except 295F), 295W, 295X, V11A | F200–F209 (except F207), F231, F232, F250–F252, F258, F259 | - |
| MDD | 300.4 | 296B, 300E, 311 | F32.0, F32.1, F32.2, F32.3, F32.8, F32.9, F33.0, F33.1, F33.2, F33.3, F33.4, F33.8, F33.9, F34.8, F34.9, F38.0, F38.1, F38.8, F39 | - |
| OCD | 300.3 | 300D | F42.0, F42.1, F42.2, F42.8, F42.9 | - |
| Any anxiety disorder | 300.0, 300.2 | 300A, 300C | F40.0, F40.1, F40.2, F40.8, F40.9, F41.0, F41.1, F41.2, F41.3, F41.8, F41.9 | - |
| ASD | - | 299A | F84.0, F84.1, F84.5 | - |
| ADHD | - | 314 | F90.0, F90.1, F90.8, F90.9 | - |
| SUD | 303, 304 | 303A, 303X, 304A-304H, 304W, 304X, 305A, 305B, 305X, | F10-F19 | - |
| Suicide attempts | E950-E959, E980-E989 | E950-E959, E980-E989 | X60-X84, Y10-Y34 | - |
| Abbreviations: ED=eating disorder; AN=anorexia nervosa; OED=other eating disorders; OCD=obsessive-compulsive disorder; MDD=major depressive disorder; ASD=autism spectrum disorder; ADHD=attention-deficit/hyperactivity disorder; SUD= substance abuse disorders.  ^a^ Diagnoses in the ED quality registers were additionally validated by structured clinical interviews (de Man Lapidoth & Birgegård, 2010). | | | | |

| **Table S2. Descriptive characteristics of individuals with comorbid schizophrenia and EDs** | | |
| --- | --- | --- |
|  | AN (*N*=70) | OED (*N*=133) |
| Sex | | |
| *Female (%)* | 60 (85.7) | 111 (83.5) |
| *Male (%)* | 10 (14.3) | 22 (16.5) |
| Birth year (mean±*SD*) | 1983.0±4.4 | 1984.4±4.5 |
| Family history of schizophrenia (%) |  |  |
| *1^st^ degree relatives* | <5 | 12 (9.0) |
| *Any relative* | 8 (11.4) | 25 (18.8) |
| Age at first AN/OED diagnosis (mean±*SD*) | 19.4±4.7 | 20.88±4.7 |
| Age at first schizophrenia diagnosis | 23.26±4.10 | 22.97±4.47 |
| ED diagnosis preceding that of schizophrenia (%) | 57 (81.4) | 93 (69.9) |
| BMI (mean±*SD*) ^a^ | 18.1±3.14 | 20.21±6.07 |
| GAF score (mean±*SD*) ^b^ | 42.78±8.97 | 43.59±9.17 |
| EDE-Q scores (mean±*SD*) ^c^ | | |
| Restraint | 2.32±1.94 | 2.53±1.57 |
| Eating concern | 1.96±1.60 | 2.33±1.37 |
| Weight concern | 2.24±2.57 | 3.13±2.24 |
| Shape concern | 2.58±2.77 | 3.62±2.39 |
| Global score | 2.24±2.05 | 2.88±1.72 |
| Psychiatric comorbidities (%) |  |  |
| Anxiety | 46 (65.7) | 86 (64.7) |
| OCD | 10 (14.3) | 26 (19.5) |
| MDD | 46 (65.7) | 97 (72.9) |
| ASD | 18 (25.7) | 32 (24.1) |
| ADHD | 9 (12.9) | 23 (17.3) |
| SUD | 39 (55.7) | 60 (45.1) |
| Total follow-up years | 1703.5 | 3034.9 |
| Total diagnoses | 10 410 | 18 299 |
| Total unique diagnoses | 3158 | 5816 |
| Total suicide attempts | 659 | 1040 |
| ^a^ There were 19 individuals with AN with BMI information, and 24 individuals with OED with BMI information.  ^b^ There were 18 individuals with AN with GAF score information, and 22 individuals with OED with GAF score information.  ^c^ There were 5 individuals with AN with EDEQ information, and 9 individuals with OED with EDE-Q information.  Abbreviations: ED=eating disorder; AN=anorexia nervosa; OED=other eating disorders; SD=standard deviation; BMI=body mass index; GAF scores= global assessment of functioning scores; EDE-Q scores=eating disorders examination questionnaire scores; OCD=obsessive-compulsive disorder; MDD=major depressive disorder; ASD=autism spectrum disorder; ADHD=attention deficit hyperactivity disorder; SUD= substance abuse disorders. | | |

| **Table S3. The prevalence (%) of psychiatric comorbidities among individuals with EDs** | | | | | |
| --- | --- | --- | --- | --- | --- |
|  |  | 1^st^ degree relatives with schizophrenia | | Any relative with schizophrenia | |
|  |  | Yes (*N*=94) | No (*N*=12 330) | Yes (*N*=599) | No (*N*=11 825) |
| AN | Anxiety | 41.5 | 27.6 | 31.9 | 27.5 |
|  | MDD | 45.7 | 35.5 | 40.1 | 35.4 |
|  | OCD | 8.5 | 7.4 | 7.2 | 7.4 |
|  | ASD | 6.4 | 3.4 | 4.0 | 3.4 |
|  | ADHD | 12.8 | 5.5 | 6.0 | 5.5 |
|  | SUD | 23.4 | 11.5 | 15.7 | 11.3 |
|  |  | Yes (*N*=199) | No (*N*=20 517) | Yes (*N*=1118) | No (*N*=19 598) |
| OED | Anxiety | 48.2 | 36.2 | 40.9 | 36.0 |
|  | MDD | 49.7 | 42.9 | 46.5 | 42.7 |
|  | OCD | 12.6 | 7.4 | 7.5 | 7.4 |
|  | ASD | 6.0 | 4.0 | 4.9 | 4.0 |
|  | ADHD | 13.6 | 8.8 | 10.2 | 8.7 |
|  | SUD | 23.1 | 15.1 | 17.2 | 15.1 |
| Abbreviations: ED=eating disorder; AN=anorexia nervosa; OED=other eating disorders; OCD=obsessive-compulsive disorder; MDD=major depressive disorder; ASD=autism spectrum disorder; ADHD=attention-deficit/hyperactivity disorder; SUD= substance abuse disorders. | | | | | |

| **Table S4. The order of first diagnosis between ED and psychiatric comorbidities** | | | |
| --- | --- | --- | --- |
|  |  | No. of individuals-before ED (%) | No. of individuals-after ED (%) |
| AN | SCZ | 13 (18.57) | 57 (81.43) |
|  | Anxiety | 1139 (33.06) | 2306 (66.94) |
|  | MDD | 1631(36.85) | 2795 (63.15) |
|  | OCD | 271 (29.36) | 652 (70.64) |
|  | ASD | 128 (29.77) | 302 (70.23) |
|  | ADHD | 187 (27.30) | 498 (72.70) |
|  | SUD | 507 (35.33) | 928 (64.67) |
| OED | SCZ | 40 (30.07) | 93 (69.92) |
|  | Anxiety | 3792 (50.46) | 3723 (49.54) |
|  | MDD | 4945 (55.59) | 3950 (44.41) |
|  | OCD | 729 (47.31) | 812 (52.69) |
|  | ASD | 327 (39.02) | 511 (60.98) |
|  | ADHD | 647 (35.43) | 1179 (64.57) |
|  | SUD | 1514 (48.16) | 1630 (51.84) |
| Abbreviations: ED=eating disorder; AN=anorexia nervosa; OED=other eating disorders; OCD=obsessive-compulsive disorder; MDD=major depressive disorder; ASD=autism spectrum disorder; ADHD=attention-deficit/hyperactivity disorder; SUD= substance abuse disorders. | | | |

| **Table S5. The hazard ratios (HRs) of psychiatric comorbidities among individuals with EDs with schizophrenia family history according to degree of relatedness.** | | | | | | | |
| --- | --- | --- | --- | --- | --- | --- | --- |
|  |  | 1^st^ degree relatives with schizophrenia | | 2^nd^ degree relatives with schizophrenia | | 3^rd^ degree relatives with schizophrenia | |
| ED types |  | HR | 95%CI | HR | 95%CI | HR | 95%CI |
| AN | Anxiety | **1.47** | **[1.08, 2.01]** | 1.14 | [0.95,1.37] | 0.96 | [0.70, 1.31] |
|  | MDD | 1.29 | [0.95, 1.75] | 1.03 | [0.88,1.22] | 1.08 | [0.83, 1.41] |
|  | OCD | 1.01 | [0.51, 2.01] | 0.93 | [0.63, 1.35] | 0.71 | [0.35, 1.43] |
|  | ASD | 1.67 | [0.75, 3.71] | 1.31 | [0.82, 2.10] | 1.73 | [0.18, 1.82] |
|  | ADHD | **2.25** | **[1.27, 3.98]** | 1.09 | [0.72, 1.63] | 0.50 | [0.18, 1.34] |
|  | SUD | **1.93** | **[1.26, 2.98]** | 1.26 | [0.97, 1.63] | 0.98 | [0.62, 1.55] |
| OED | Anxiety | **1.36** | **[1.11, 1.67]** | 1.11 | [0.98, 1.25] | 1.06 | [0.87, 1.29] |
|  | MDD | 1.18 | [0.96, 1.44] | 1.10 | [0.98, 1.23] | 1.06 | [0.88, 1.27] |
|  | OCD | **1.59** | **[1.08, 2.36]** | 0.88 | [0.66, 1.17] | 0.76 | [0.44, 1.30] |
|  | ASD | 1.48 | [0.83, 2.63] | 1.37 | [0.99, 1.88] | 0.85 | [0.42, 1.73] |
|  | ADHD | **1.59** | **[1.09, 2.31]** | 1.15 | [0.91, 1.46] | 0.99 | [0.64, 1.53] |
|  | SUD | **1.45** | **[1.08, 1.96]** | 1.08 | [0.90, 1.30] | 0.91 | [0.66, 1.25] |
| Abbreviations: ED=eating disorder; CI= confidence interval; AN=anorexia nervosa; OED=other eating disorders; OCD=obsessive-compulsive disorder; MDD=major depressive disorder; ASD=autism spectrum disorder; ADHD=attention-deficit/hyperactivity disorder; SUD= substance abuse disorders.  **Bold font** indicates statistical significance, p<0.05. | | | | | | | |

| **Table S6. The incidence rate ratios (IRRs) of cumulative somatic and mental health burden among individuals with EDs with schizophrenia family history according to degree of relatedness.** | | | | | | | |
| --- | --- | --- | --- | --- | --- | --- | --- |
|  |  | 1^st^ degree relatives with schizophrenia | | 2^nd^ degree relatives with schizophrenia | | 3^rd^ degree relatives with schizophrenia | |
| ED types |  | IRR | 95%CI | IRR | 95%CI | IRR | 95%CI |
| AN | Total diagnoses | 1.11 | [0.88, 1.42] | 1.14 | [0.98,1.34] | 1.15 | [0.87, 1.53] |
|  | Total unique diagnoses | 1.18 | [0.96, 1.46] | **1.13** | **[1.00,1.27]** | 1.00 | [0.84, 1.19] |
|  | Suicide attempts | 1.61 | [0.83, 3.14] | 1.32 | [0.77, 2.24] | 1.08 | [0.41, 2.81] |
| OED | Total diagnoses | **1.26** | **[1.07, 1.48]** | 1.10 | [0.98,1.22] | 1.01 | [0.84, 1.21] |
|  | Total unique diagnoses | **1.24** | **[1.08, 1.41]** | 1.08 | [1.00,1.16] | 0.96 | [0.85, 1.09] |
|  | Suicide attempts | **1.70** | **[1.05, 2.76]** | 1.08 | [0.71, 1.66] | 1.09 | [0.84, 1.19] |
| Abbreviations: ED=eating disorder; CI= confidence interval; AN=anorexia nervosa; OED=other eating disorders.  **Bold font** indicates statistical significance, p<0.05. | | | | | | | |

| **Table S7. The hazard ratios (HRs) of psychiatric comorbidities among individuals with EDs with schizophrenia family history (before ED diagnosis).** | | | | | |
| --- | --- | --- | --- | --- | --- |
|  |  | 1^st^ degree relatives with schizophrenia | | Any relative with schizophrenia | |
| ED types |  | HR | 95%CI | HR | 95%CI |
| AN | Anxiety | 1.14 | [0.64, 2.02] | 1.10 | [0.84, 1.45] |
|  | MDD | **1.60** | **[1.02, 2.51]** | 1.15 | [0.92, 1.43] |
|  | OCD | NA^a^ | NA^a^ | 0.84 | [0.46, 1.54] |
|  | ASD | 0.75 | [0.11, 5.36] | 1.20 | [0.55, 2.58] |
|  | ADHD | 2.32 | [0.75, 7.20] | 1.05 | [0.54, 2.04] |
|  | SUD | **2.07** | **[1.05, 4.05]** | 1.39 | [0.99, 1.96] |
| OED | Anxiety | **1.47** | **[1.11, 1.94]** | 1.14 | [1.00, 1.30] |
|  | MDD | **1.36** | **[1.06, 1.75]** | 1.12 | [1.00, 1.26] |
|  | OCD | 1.37 | [0.74, 2.52] | 0.92 | [0.66, 1.27] |
|  | ASD | 1.34 | [0.49, 3.66] | 1.34 | [0.87, 2.06] |
|  | ADHD | **2.19** | **[1.20, 4.02]** | 1.15 | [0.83, 1.60] |
|  | SUD | **1.74** | **[1.17, 2.58]** | 1.10 | [0.89, 1.36] |
| Abbreviations: ED=eating disorder; CI= confidence interval; AN=anorexia nervosa; OED=other eating disorders; OCD=obsessive-compulsive disorder; MDD=major depressive disorder; ASD=autism spectrum disorder; ADHD=attention deficit hyperactivity disorder; SUD= substance abuse disorders.  **Bold font** indicates statistical significance, p<0.05.  a: There was no outcome in the exposed group. | | | | | |

| **Table S8. The hazard ratios (HRs) of psychiatric comorbidities among individuals with eating disorders with schizophrenia family history (after ED diagnosis).** | | | | | |
| --- | --- | --- | --- | --- | --- |
|  |  | 1^st^ degree relatives with schizophrenia | | Any relative with schizophrenia | |
| ED types |  | HR | 95%CI | HR | 95%CI |
| AN | Anxiety | **1.66** | **[1.14, 2.40]** | 1.14 | [0.96, 1.36] |
|  | MDD | 1.16 | [0.76, 1.76] | 1.11 | [0.95, 1.31] |
|  | OCD | 1.50 | [0.75, 2.99] | 0.94 | [0.66, 1.34] |
|  | ASD | 2.18 | [0.92, 5.16] | 1.09 | [0.67, 1.77] |
|  | ADHD | **2.07** | **[1.42, 3.03]** | 1.04 | [0.71, 1.54] |
|  | SUD | **1.82** | **[1.03, 3.23]** | 1.24 | [0.95, 1.62] |
| OED | Anxiety | 1.24 | [0.92, 1.67] | 1.10 | [0.96, 1.26] |
|  | MDD | 0.98 | [0.72, 1.34] | 1.06 | [0.93, 1.21] |
|  | OCD | **1.82** | **[1.09, 3.03]** | 1.02 | [0.75, 1.37] |
|  | ASD | 1.57 | [0.77, 3.20] | 1.19 | [0.83, 1.70] |
|  | ADHD | 1.35 | [0.83, 2.20] | 1.19 | [0.95, 1.50] |
|  | SUD | 1.23 | [0.79, 1.92] | 1.08 | [0.88, 1.32] |
| Abbreviations: ED=eating disorder; CI= confidence interval; AN=anorexia nervosa; OED=other eating disorders; OCD=obsessive-compulsive disorder; MDD=major depressive disorder; ASD=autism spectrum disorder; ADHD=attention deficit hyperactivity disorder; SUD= substance abuse disorders.  **Bold font** indicates statistical significance, p<0.05. | | | | | |

| **Table S9. The incidence rate ratios (IRRs) of cumulative somatic and mental health burden among individuals with eating disorders with schizophrenia family history (before ED diagnosis).** | | | | | |
| --- | --- | --- | --- | --- | --- |
|  |  | 1^st^ degree relatives with schizophrenia | | Any relative with schizophrenia | |
| ED types |  | IRR | 95%CI | IRR | 95%CI |
| AN | Total diagnoses | 1.05 | [0.80, 1.40] | 1.04 | [0.91,1.20] |
|  | Total unique diagnoses | 1.19 | [0.96, 1.47] | **1.12** | **[1.00,1.25]** |
|  | Total suicide attempts | 1.04 | [0.33, 3.25] | 1.01 | [0.57, 1.78] |
| OED | Total diagnoses | 1.15 | [0.88, 1.51] | 1.06 | [0.95, 1.19] |
|  | Total unique diagnoses | 1.12 | [0.86, 1.45] | 1.02 | [0.94, 1.12] |
|  | Total suicide attempts | **2.14** | **[1.04, 4.43]** | 1.24 | [0.84, 1.83] |
| Abbreviations: ED=eating disorder; CI= confidence interval; AN=anorexia nervosa; OED=other eating disorders.  **Bold font** indicates statistical significance, p<0.05. | | | | | |

| **Table S10. The incidence rate ratios (IRRs) of cumulative somatic and mental health burden among individuals with eating disorders with schizophrenia family history (after ED diagnosis).** | | | | | |
| --- | --- | --- | --- | --- | --- |
|  |  | 1^st^ degree relatives with schizophrenia | | Any relative with schizophrenia | |
| ED types |  | IRR | 95%CI | IRR | 95%CI |
| AN | Total diagnoses | 1.12 | [0.82, 1.53] | 1.11 | [0.95,1.30] |
|  | Total unique diagnoses | 1.17 | [0.87, 1.56] | 1.05 | [0.93,1.20] |
|  | Total suicide attempts | 1.75 | [0.90, 3.40] | 1.42 | [0.92, 2.20] |
| OED | Total diagnoses | 1.14 | [0.92, 1.42] | 1.06 | [0.94, 1.20] |
|  | Total unique diagnoses | 1.12 | [0.93, 1.36] | 1.05 | [0.96, 1.16] |
|  | Total suicide attempts | 1.22 | [0.69, 2.17] | 0.99 | [0.68, 1.43] |
| Abbreviations: ED=eating disorder; CI= confidence interval; AN=anorexia nervosa; OED=other eating disorders. | | | | | |

| **Table S11. The incidence rate ratios (IRRs) of cumulative somatic and mental health burden among individuals with eating disorders with schizophrenia family history (comorbid diagnoses observed together with ED diagnoses).** | | | | | |
| --- | --- | --- | --- | --- | --- |
|  |  | 1^st^ degree relatives with schizophrenia | | Any relative with schizophrenia | |
| ED types |  | IRR | 95%CI | IRR | 95%CI |
| AN | Total diagnoses | 0.85 | [0.52, 1.42] | 1.32 | [0.99, 1.76] |
|  | Total unique diagnoses | 1.14 | [0.78, 1.67] | **1.28** | **[1.07, 1.53]** |
| OED | Total diagnoses | 1.01 | [0.75, 1.36] | 1.09 | [0.92, 1.28] |
|  | Total unique diagnoses | 1.22 | [0.98, 1.51] | 1.06 | [0.96, 1.17] |
| Abbreviations: ED=eating disorder; CI= confidence interval; AN=anorexia nervosa; OED=other eating disorders.  **Bold font** indicates statistical significance, p<0.05. | | | | | |

**Reference**

de Man Lapidoth, J., & Birgegård, A. (2010). Validation of the structured eating disorder interview (SEDI) against the eating disorder examination (EDE). *Stockholm: Karolinska Institutet*.
